# The Involvement of the microRNAs miR-466c and miR-340 in the Palmitate-Mediated Dysregulation of Gonadotropin-Releasing Hormone Gene Expression

**DOI:** 10.3390/genes15040397

**Published:** 2024-03-23

**Authors:** Vanessa Nkechika, Ningtong Zhang, Denise D. Belsham

**Affiliations:** 1 Department of Physiology, University of Toronto, 1 King’s College Circle, Toronto, ON M5S 1A8, Canada; vanessa.nkechika@mail.utoronto.ca (V.N.); ningtong.zhang@mail.utoronto.ca (N.Z.); 2 Department of Medicine, University of Toronto, 1 King’s College Circle, Toronto, ON M5S 1A8, Canada

**Keywords:** obesity, palmitate, hypothalamus, GnRH, microRNA, transcription factors

## Abstract

Diets high in saturated fatty acids are associated with obesity and infertility. Palmitate, the most prevalent circulating saturated fatty acid, is sensed by hypothalamic neurons, contributing to homeostatic dysregulation. Notably, palmitate elevates the mRNA levels of gonadotropin-releasing hormone (*Gnrh*) mRNA and its activating transcription factor, GATA binding protein 4 (*Gata4*). GATA4 is essential for basal *Gnrh* expression by binding to its enhancer region, with Oct-1 (*Oct1*) and CEBP-β (*Cebpb*) playing regulatory roles. The pre- and post-transcriptional control of *Gnrh* by palmitate have not been investigated. Given the ability of palmitate to alter microRNAs (miRNAs), we hypothesized that palmitate-mediated dysregulation of *Gnrh* mRNA involves specific miRNAs. In the mHypoA-GnRH/GFP neurons, palmitate significantly downregulated six miRNAs (miR-125a, miR-181b, miR-340, miR-351, miR-466c and miR-503), and the repression was attenuated by co-treatment with 100 μM of oleate. Subsequent mimic transfections revealed that miR-466c significantly downregulates *Gnrh*, *Gata4*, and *Chop* mRNA and increases *Per2*, whereas miR-340 upregulates *Gnrh, Gata4*, *Oct1*, *Cebpb*, and *Per2* mRNA. Our findings suggest that palmitate may indirectly regulate *Gnrh* at both the pre- and post-transcriptional levels by altering miR-466c and miR-340, which in turn regulate transcription factor expression levels. In summary, palmitate-mediated dysregulation of *Gnrh* and, consequently, reproductive function involves parallel transcriptional mechanisms.

## 1. Introduction

The mechanistic regulation of reproductive function by diet is not fully understood. It is, however, understood that diets rich in saturated fatty acids have negative effects on the regulation of the reproductive axis and thus fertility. In contrast, diets enriched in unsaturated fatty acids are associated with better reproductive health [1]. The rise in high-fat diet (HFD)-induced obesity has led to an increased occurrence of obesity-related comorbidities, including infertility. Notably, the risk of infertility increases by 78% by childbearing age in obese women compared to their normal-BMI counterparts [2]. It is thus imperative that the effects of HFD on reproductive health are studied.

The hypothalamic–pituitary–gonadal axis (HPG axis) is the regulatory system of reproduction and sexual development. It consists of the hypothalamus, anterior pituitary gland, and the gonads. Regulated through a negative feedback loop, *Gnrh* neurons of the hypothalamus secrete gonadotropin-releasing hormone in a pulsatile manner into the anterior pituitary gland, stimulating the release of luteinizing hormone (LH) and follicle-stimulating hormone (FSH). These tropic hormones lead to the production of testosterone in men and estrogen and progesterone in women by their respective gonads [3]. *Gnrh* transcription is controlled by stimulatory and inhibitory transcription factors that bind to its promoter and enhancer regions [4]. Mutations or deletions of these essential transcription factors result in the attenuation of basal *Gnrh* expression in the hypothalamus. One such factor is the GATA4 [5], which binds to the proximal enhancer alongside Oct-1 and CEBP-β, both involved in nitric oxide- and melatonin-mediated repression of *Gnrh* mRNA. The *Gnrh* enhancer contains two GATA binding motifs, GATA-A and GATA-B, with GATA-B being preferentially involved in enhancer-specific activation [5]. The regulation of *Gnrh* expression is a complex interplay of these transcription factors, with GATA4 playing a significant role in the generation and activation of GnRH neurons [5].

Palmitate, a 16-carbon chain saturated fatty acid (C16:0), is the most prevalent fatty acid in the human body. It is increased after the consumption of an HFD or synthesized endogenously from other fatty acids, carbohydrates, and amino acids [6]. Palmitate is important for basal functions of the cell; however, excessive consumption, as seen in obese individuals, can lead to detrimental effects. The hypothalamus can sense these elevated circulating free fatty acids [7,8], which are associated with dysregulation in neuroendocrine signaling, circadian clock dysfunction, neuroinflammation, and endoplasmic reticulum stress [6]. Our laboratory has studied the effects of palmitate on many levels, including signal transduction events, detrimental cellular functions, and transcriptionally [6]. A study by Tran et al. demonstrated the upregulation of *Gnrh* mRNA by palmitate in the immortalized mHypoA-GnRH/GFP cell line [9]. Mechanistically, it was determined that the palmitate-mediated increase in *Gnrh* mRNA partially involves upstream PI3K signaling; however, it remains unknown whether palmitate can regulate *Gnrh* mRNA at the pre- or post-transcriptional levels.

MicroRNAs (miRNAs) are evolutionarily conserved post-transcriptional gene regulators. These small noncoding RNAs (~22 nucleotides) bind to the 3’ untranslated regions (UTR) of genes, inhibiting translation or causing the mRNA degradation of target genes [10,11]. Clinical studies demonstrate a difference in miRNA profiles between obese and non-obese individuals, possibly linking the involvement of miRNAs in the pathogenesis of obesity [12,13,14,15,16]. Moreover, miRNAs in the hypothalamus control facets of energy homeostasis [17,18]. The deletion of *Dicer* in the ARC of mice leads to miRNA deficiency, resulting in altered neuropeptide expression and obesity [18]. Hypothalamic miRNAs also play a crucial role in regulating reproductive function, and disturbances in this regulation can lead to reproductive disorders [19]. For example, miR-146a, miR-155, and miR-486 are upregulated in the granulosa cells of women with polycystic ovary syndrome (PCOS), while miR-148a is downregulated in women with endometritis [19]. In other models, palmitate has been linked to the dysregulation of cellular miRNA profiles, leading to disrupted metabolic homeostasis, an effect that is alleviated by oleate [20]. Based on these observations, we hypothesized that the palmitate-mediated dysregulation of *Gnrh* mRNA is mediated by post-transcriptional regulation through miRNAs and that oleate may counteract the effects of palmitate. This study aimed to identify the miRNA targets of *Gnrh* in the mHypoA-GnRH/GFP murine cell line, using a curated list of predicted miRNA targets of *Gnrh*.

We describe the fact that palmitate alters the expression of activating transcription factor *Gata4* and miRNAs (miR-340, miR-125a, miR-181b, miR-503, miR-351, and miR-466c) that are predicted to target the *Gnrh* mRNA. Furthermore, co-treatment with oleate mitigates this palmitate-mediated action. Overall, we implicate the involvement of miRNAs in the palmitate-mediated dysregulation of *Gnrh* and its associated transcription factors, ultimately affecting reproductive control at the hypothalamic level.

## 2. Methods

### 2.1. Cell Culture and Reagent Preparation

The mHypoA-GnRH/GFP cell line, and the array comparative cell lines mHypoE-46 and mHypoA-59, were generated and characterized as previously described [9,21]. The main model used in this study was the mHypoA-GnRH/GFP that was previously reported to sense fatty acids [9]. mHypoA-GnRH/GFP cells were cultured in low-glucose (5.5 mM) Dulbecco’s modified eagle medium (DMEM), supplemented with 5% fetal bovine serum (FBS) and 1% penicillin–streptomycin (P/S) in a 37 °C incubator with 5% CO_2_. Cells were plated into 60 mm tissue culture plates and grown to 60–75% confluency for 24 h. Before 100 μM palmitate and/or oleate treatments, cells underwent serum starvation for 2 h in FBS-free DMEM, followed by synchronization with a 1 h serum shock using 5% FBS and 20 μM forskolin.

Sodium palmitate and sodium oleate were dissolved in ultra-pure distilled H_2_O to 100 mM by heating at 70 °C. These stock solutions were diluted 1:1000 in treatment media (5% FBS-containing DMEM) to achieve final treatment concentrations of 100 μM palmitate or oleate. Ultra-pure distilled H_2_O served as the vehicle control. Cells were treated with 100 μM of palmitate, 100 μM of oleate, or a combination of both for 24 h before cell lysis and RNA isolation.

### 2.2. RNA Isolation and RT-qPCR

Cells were harvested using the Norgen lysis buffer (RL) and total RNA was isolated using the Norgen Total RNA Purification kit (Norgen Biotek Corp., Thorold, ON, Canada) and on-column DNase (Norgen Biotek Corp.). The quantity and purity of the RNA were analyzed using the Nanodrop2000 spectrophotometer (Thermofisher Scientific, Mississauga, ON, Canada) and complimentary DNA (cDNA) synthesized by reverse transcription (Applied Biosystems, Mississauga, ON, Canada). cDNA was amplified using quantitative reverse transcriptase polymerase chain reaction (qRT-PCR) and Powertrack SYBR green master mix with gene-specific primers (Table 1), as described previously [21]. qRT-PCR results were analyzed using the ∆∆Ct method and normalized to a reference gene (ribosomal protein L7 (*Rpl7*)), respectively.

cDNA for miRNA analysis was synthesized using 100 ng in a 10 uL reaction containing 1 μL of 10× reaction buffer *E. coli* poly(A) polymerase, 1 μL of 1 mM of ATP, 1 μL of 10 μM RT primer 5′-CAGGTCCAGTTTTTTTTTTTTTTTVN, 2 μL (50 U/uL) of MultiScribe™ reverse transcriptase, 0.2 μL of *E. coli* poly (A) polymerase (5000 U/mL), and 1 μL of dNTP mix (1 mM dATP, 1 mM dCTP, 1 mM dGTP, 1 mM dTTP). cDNA was prepared using the miRCURY heating protocol (Qiagen). miRNA expression levels were quantified by RT-qPCR using gene-specific primers (Table 2), as described previously [21]. The results were analyzed using the ∆∆Ct method and normalized to reference microRNA- mmu-miR-221-3p. The protocol was adapted from Balcells et al., 2011 [22].

### 2.3. MicroRNA Mimic Transfections

The mHypoA-GnRH/GFP cells were grown to 70–80% confluency in 60 mm tissue culture plates for 24 h transfections. An amount of 25 nM of the mirVana miRNA mimics (Thermofisher Scientific) or negative control was complexed for 20 min with Dharmafect 3 transfection reagent (Dharmacon, Cedarlane, Burlington, ON, Canada) at room temperature in low-glucose (5.5 mM) DMEM without FBS and P/S. The complex (500 μL) was added onto the mHypoA-GnRH/GFP cells cultured in 2 mL of 2% FBS DMEM (no P/S) for 24 h.

### 2.4. Statistical Analysis

The RT-qPCR results were statistically analyzed using GraphPad Prism 9.0.2 software (GraphPad Software Inc., San Diego, CA, USA). Each experiment was conducted with at least *n* = 3 biological replicates, as in the figure legends. A Student *t*-test or a two-way ANOVA with post hoc Tukey HSD for all treatment groups was performed to determine significant changes, where *p* < 0.05. All data are presented as mean ± SEM. Statistical significance is denoted by * *p* < 0.05, ** *p* < 0.01, *** *p* < 0.001, **** *p* < 0.0001. The exact values are indicated in Table 3.

**Table 3:** Changes in expression of indicated genes and miRNAs.

	Gene/microRNA	Treatment	Fold Change	SEM	*p* Value
Figure 1	*Gnrh*	−	1.14	0.2396	** 0.0028
		+	−1.07	0.2588	** 0.0079
	*Gata4*	−	0.49	0.128	* 0.0127
		+	−0.52	0.1382	* 0.0143
	*Chop*	−	2.93	0.4407	*** 0.0002
		+	−2.39	0.4761	** 0.0019
	*Per2*	−	−0.69	0.1806	* 0.0205
		+	1.15	0.1806	*** 0.0010
Figure 2	*miR-125a*	−	−0.55	0.1176	** 0.0025
		+	0.45	0.1176	* 0.0117
	*miR-181b*	−	−0.54	0.108	** 0.0015
		+	0.61	0.108	*** 0.0006
	*miR-351*	−	−0.62	0.1904	* 0.0307
		+	0.94	0.1904	** 0.0018
	*miR-340*	−	−0.63	0.161	** 0.0097
		+	0.73	0.161	** 0.0034
	*miR-466c*	−	−0.42	0.05808	**** <0.0001
		+	0.34	0.05808	*** 0.0004
	*miR-503*	−	−0.61	0.1408	** 0.0046
		+	0.78	0.1408	*** 0.0006
Figure 3A	*Gnrh*	25 nM of miR-466c	−0.14	0.03007	** 0.0032
		25 nM of miR-340	0.32	0.09319	** 0.0088
Figure 4A	*Gata4*	25 nM of miR-466c	−0.34	0.1058	* 0.0179
	*Chop*		−0.2	0.01704	**** <0.0001
	*Per2*		0.8	0.09364	** 0.0010
Figure 4B	*Cebpb*	25 nM of miR-340	0.31	0.04581	*** 0.0002
	*0ct1*		0.17	0.05706	* 0.0157
	*Gata4*		0.25	0.08935	* 0.0225
	*Per2*		0.46	0.02114	**** <0.0001
Figure 5	*Gnrh*	100 µM of palmitate + NC	0.56	0.1526	* 0.0309
		100 µM of palmitate + 25 nM of miR-340	0.69	0.1526	** 0.0074

NB: 100 µM of palmitate (−); 100 µM of palmitate + 100 µM of oleate (+).

**Figure 1:**
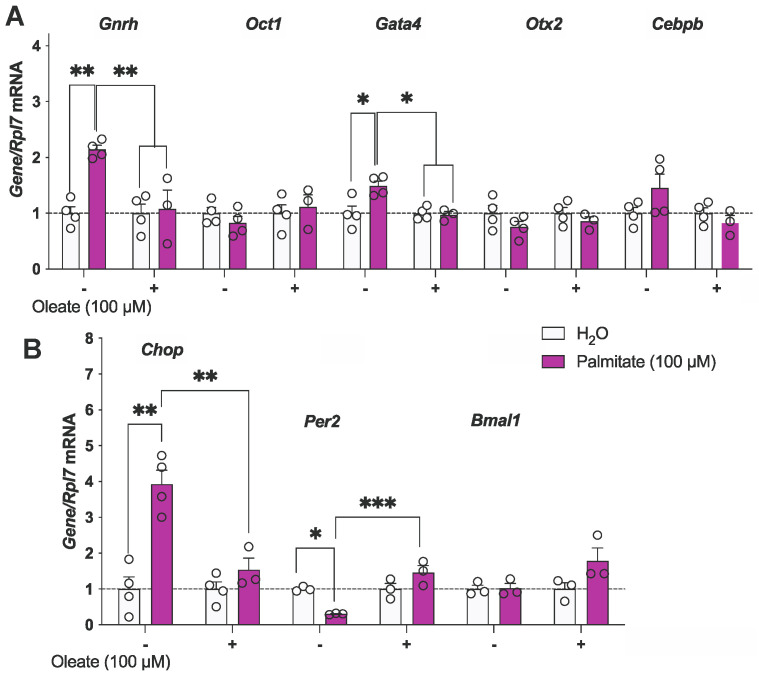
Oleate protects against palmitate-mediated alterations of *Gnrh*, *Gata4, and Per2* mRNA expression. (**A**,**B**) mHypoA-GnRH/GFP cells were treated with either vehicle (H_2_O), 100 μM of palmitate, 100 μM of oleate, or both 100 μM of palmitate and 100 μM of oleate for 24 h. Results are expressed as mean ± SEM. *n* = 3–4. Statistical significance determined using a two-way ANOVA, followed by the Tukey’s multiple comparison test: * *p* < 0.05, ** *p* < 0.01, *** *p* < 0.001.

**Figure 2:**
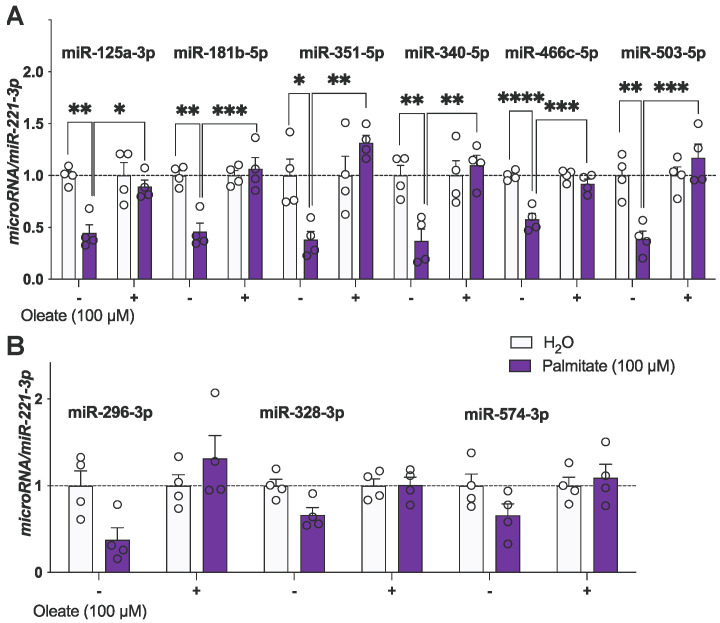
Palmitate downregulates microRNAs predicted to target *Gnrh*. (**A**,**B**) mHypoA-GnRH/GFP cells were treated with either vehicle (H_2_O), 100 μM of palmitate, 100 μM of oleate, or 100 μM of palmitate and 100 μM of oleate for 24 h. Results are expressed as mean ± SEM. *n* = 4. Statistical significance determined using a two-way ANOVA, followed by the Tukey’s multiple comparison test: * *p* < 0.05, ** *p* < 0.01, *** *p* < 0.001, **** *p* < 0.0001.

**Figure 3:**
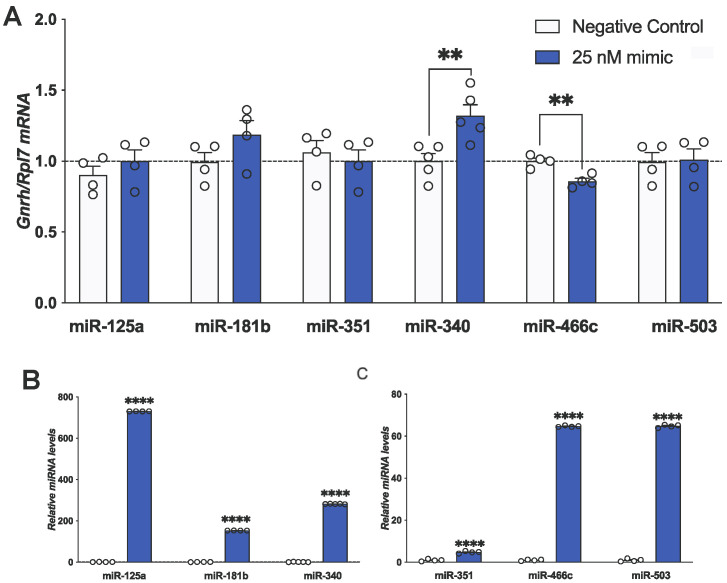
miR-466c and miR-340 alter *Gnrh* mRNA expression. mHypoA-GnRH/GFP neurons were treated with either 25 nM of mimic or negative control for 24 h. (**A**) *Gnrh* expression or (**B**,**C**) miRNA expression was quantified using qPCR. Results are expressed as mean ± SEM. *n* = 4–5. Statistical significance was determined using Student’s *t*-test (two-tailed, unpaired, equal variance assumed). ** *p* < 0.01, **** *p* < 0.0001.

**Figure 4:**
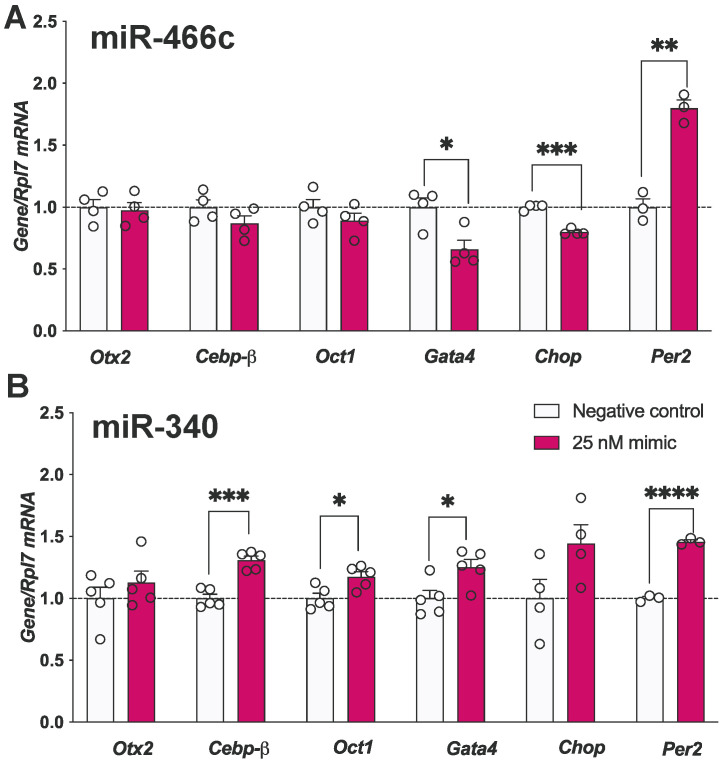
miR-466c upregulates *Per2* and downregulates *Chop* and *Gata4* expression, while miR-340 upregulates *Cebpb*, *Gata4* and *Oct1.* mHypoA-GnRH/GFP neurons were treated with (**A**) 25 nM of miR-466c mimic or (**B**) 25 nM of miR-340 mimic alongside negative control for 24 h. Results are expressed as mean ± SEM. *n* = 4–5. Statistical significance determined using Student’s *t*-test (two-tailed, unpaired, equal variance assumed): * *p* < 0.05, ** *p* < 0.01, *** *p* < 0.001, **** *p* < 0.0001.

**Figure 5:**
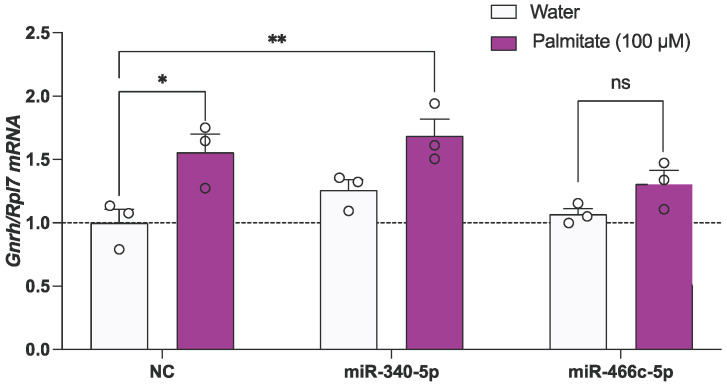
miR-466c mitigates the palmitate-mediated upregulation of *Gnrh* in mHypoA-GnRH/GFP neurons. mHypoA-GnRH/GFP neurons were treated with 25 nM of miR-466c mimic or miR-340 mimic alongside negative control for 24 h. After 24 h, neurons were treated with either vehicle (H_2_O) or 100 μM of palmitate for 24 h. Results are expressed as mean ± SEM. *n* = 3. Statistical significance determined using a two-way ANOVA, followed by the Tukey’s multiple comparison test: * *p* < 0.05, ** *p* < 0.01.

## 3. Results

### 3.1. Oleate Protects against Palmitate-Mediated Upregulation of Gnrh mRNA Expression

Consistent with our previously published findings [9], we observed an increase in *Gnrh* mRNA levels following 24 h of treatment with 100 μM of palmitate in the mHypoA-GnRH/GFP cell line. The palmitate-mediated upregulation was attenuated by a 24 h 100 μM oleate co-treatment (Figure 1A). The mRNA expression of previously described transcription factors binding the *GnRH* enhancer, activating (*Oct1*, *Gata4*, *Otx2*) and inhibitory transcription factors (*Oct1*, *Cebpb*), was also evaluated [3]. *Gata4* mRNA levels were significantly increased following palmitate treatment, while oleate co-treatment prevented this increase (Figure 1A). However, the expression of *Otx2*, *Oct1*, and *Cebpb* remained unchanged at 24 h by both oleate and palmitate (Figure 1A). We also assessed palmitate-induced circadian dysregulation by examining the circadian genes period circadian regulator 2 (*Per2*) and basic helix–loop–helix ARNT like 1 (*Bmal1*) since circadian dysregulation is also linked to reproductive dysfunction [23]. Palmitate downregulated *Per2* (Figure 1B) but had no effect on *Bmal1*. Furthermore, the endoplasmic reticulum stress marker protein C/EBP homologous protein (*Chop*) was evaluated as a positive control for palmitate treatment (Figure 1B). Co-treatment with oleate also blocked the palmitate-induced changes in *Per2* and *Chop* (Figure 1B).

### 3.2. Analysis of miRNAs Expressed in the mHypoA-GnRH/GFP Neurons

Next, to investigate the contribution of miRNAs to the effect of palmitate on *Gnrh* mRNA expression, we curated a list of candidate miRNAs using a set of selection parameters [24,25]. Murine microRNA candidates were selected based on (i) predicted binding to *Gnrh* 3′ UTR by TargetScanMouse 7.1 and TargetScanHuman 7.1, (ii) predicted fold changes with palmitate treatment based on a microarray in the mHypoE-46 cell line (*p* < 0.05) [26], and (iii) basal expression levels in the whole hypothalamus and other hypothalamic cells lines (mHypoA-59 and mHypoE-46 cells) as detected by a microRNA microarray (Table 4). microRNA basal expression levels identified by the microarray were validated by qRT-PCR. There were nine miRNAs that were downregulated by palmitate in the mHypoE-46 cell line that were predicted to target *Gnrh* (miR-125a-3p, miR-181b-5p, miR-296-3p, miR-328-3p, miR-351-5p, miR-466c-5p, miR-503-5p, miR-574-3p and let-7k). Eight of these miRNAs were studied further as they were highly expressed. mmu-let-7k expression was low in the mHypoA-GnRH/GFP cell line according to qRT-PCR; thus, it was not analyzed further. There were five microRNAs highly conserved between mice and humans. These included miR-876-5p, miR-224-5p, miR-485-5p, miR-340-5p and mir-539-3p. As there was a possibility of these being altered in the mHypoA-GnRH/GFP cells, we included these in the analysis. From these five, only miR-340-5p was selected for further analysis according to basal levels and primer specificity (Table 4).

### 3.3. Palmitate Alters miRNA Expression in mHypoA-GnRH/GFP Cells, While Oleate Prevents Palmitate-Induced Dysregulation

To explore the role of microRNAs in palmitate-mediated action on *Gnrh* mRNA, the levels of the selected miRNAs were assessed after palmitate and oleate treatment (Figure 2A). miR-125a, miR-181b, miR-351, miR-340, miR-466c and miR-503 were significantly downregulated following treatment with 100 μM of palmitate for 24 h. Oleate exerted a protective effect, blunting the palmitate-mediated downregulation of these microRNAs. miR-296, miR-328, and miR-574 were not significantly changed following treatment with 100 μM of palmitate, 100 μM of oleate or a combination of both 100 μM of palmitate and 100 μM of oleate for 24 h (Figure 2B).

### 3.4. miR-466c Downregulates While miR-340 Upregulates Gnrh mRNA Levels in mHypoA-GnRH/GFP Neurons

Next, we questioned whether the six miRNAs that were downregulated with palmitate treatment may be involved in the post-transcriptional regulation of *Gnrh*, ultimately leading to the palmitate-mediated induction of *Gnrh*. To begin to answer this question, we investigated the individual role of each miRNA in altering *Gnrh* mRNA levels using specific miRNA mimics. mHypoA-GnRH/GFP neurons were transfected with 25 nM of miRNA mimic or negative control for 24 h. Of the six miRNAs investigated, only the miR-466c mimic downregulated *Gnrh* mRNA, while the miR-340 mimic upregulated *Gnrh* mRNA (Figure 3A). miR-503, miR-181b, miR-351 and miR-125a did not alter *Gnrh* mRNA levels after 24 h (Figure 3A). As a control, we found that the miRNAs were increased by the individual mimics after 24 h of transfection (Figure 3B,C).

### 3.5. miR-466c and miR-340 Also Alter Transcriptional Regulators of Gnrh in mHypoA-GnRH/GFP Neurons

Although the data suggest that miR-466c may be a direct negative regulator of *Gnrh* mRNA, we wanted to investigate whether the miR-466c mimic may also indirectly regulate *Gnrh* by altering the mRNA levels of its transcription factors. *Chop* and *Gata4* were significantly downregulated by the miR-466c mimic, while *Per2* was significantly upregulated (Figure 4A). As *Gata4* is a positive regulator of *Gnrh*, miR-466c may also downregulate *Gnrh* via *Gata4*. Similarly, we investigated the potential regulation of *Gnrh*-related transcription factors by the miR-340 mimic. *Cebpb*, *Oct1* and *Gata4* (Figure 4B) were significantly upregulated following 25 nM miR-340 mimic transfection compared with the negative control. The results suggest a potential indirect regulation of *Gnrh* by miR-466c and miR-340 via altering its transcription factors.

### 3.6. miR-466c Blocks the Palmitate-Mediated Upregulation of Gnrh in mHypoA-GnRH/GFP Neurons

The results of the 24 h mimic transfections indicate that both miR-340 and mir-466c regulate *Gnrh* expression independent of palmitate exposure (Figure 3A). While we established that palmitate exposure represses these miRNAs in the mHypoA-GnRH/GFP neurons (Figure 2A), it was previously unknown whether the palmitate-induced increase in *Gnrh* is mediated by these miRNAs. Thus, we pre-treated the neurons with mimics to prevent the suppression of these miRNAs by palmitate. mHypoA-GnRH/GFP cells were pre-exposed to 25 nM of miR-466c or miR-340 for 24 h, followed by treatment with 100 μM of palmitate for 24 h. miR-466c pre-exposure blocked the palmitate-mediated upregulation of *Gnrh* mRNA (Figure 5). These results suggest that the suppression of miR-466c by palmitate is necessary for the palmitate-mediated increase in *Gnrh*.

## 4. Discussion

The impact of a high-fat diet on reproductive health and the influence of palmitate on *Gnrh* expression have been previously documented [27,28]. The levels of the free fatty acids (FFAs) used in this study are well within the circulating levels in humans. The normal fasting serum FFA level is 0.1 to 0.45 mM for females and 0.1 to 0.6 mM for males. Palmitate, the most abundant FFA in the human diet and a dominant circulating saturated NEFA, has upper levels of approximately 0.1 mM. Oleate levels are in a similar range, although somewhat lower. However, on a high-fat diet, these levels can increase to mM levels, depending upon the composition of the diet [29,30,31]. In this study, we present, for the first time, the effects of palmitate on microRNAs predicted to target the 3′UTR of *Gnrh* (Table 5). Our findings suggest that palmitate may exert some of its detrimental effects by post-transcriptionally regulating *Gnrh*. Additionally, we observe that oleate, known for its protective role against palmitate-induced effects [32,33,34], also safeguards against changes in these microRNAs. This study provides compelling evidence of palmitate-mediated dysregulation of reproductive function through microRNAs, thus shedding light on a potential post-transcriptional mechanism underlying high-fat-diet-induced reproductive dysfunction.

We observed a significant downregulation of microRNAs predicted to target *Gnrh* mRNA at 24 h following palmitate treatment. microRNAs function by destabilizing mRNAs, leading to their degradation and translational inhibition [19]. As palmitate downregulates these microRNAs and concurrently allows the increase in *Gnrh*, it implies a potential regulatory role of these microRNAs in mediating palmitate-induced *Gnrh* repression. Interestingly, our results suggest the existence of microRNAs that directly regulate the *Gnrh* gene. Subsequent 24 h mimic transfections of these microRNAs reveal that not all may be involved in palmitate-mediated *Gnrh* repression, as they do not downregulate *Gnrh* mRNA following endogenous overexpression in the mHypoA-GnRH/GFP cell line. The suppression of some microRNAs by palmitate are likely independent of *Gnrh* regulation. miR-351 and miR-503, for example, are part of the miR-424/322/351/503 cluster encoded on chromosome X [35], which is found to be downregulated during the ER stress response via PERK signaling [36]. Since palmitate is known to induce ER stress, miR-503 and miR-351 downregulation may be a stress response instead of a *Gnrh*-related response. Additionally, we found a significant downregulation of miR-503 following palmitate treatment, which was blunted with oleate co-treatment in the Npy/Agrp-expressing mHypoE-46 cell line [26]. This finding strengthens the idea that the palmitate-mediated suppression of miR-503 is common across different neuronal cell lines.

The highly conserved miR-340-5p, predicted to target the human and mouse *Gnrh* gene (according to TargetScanMouse 7.1), was downregulated by palmitate. It is encoded within the intronic region of the Ring finger 130 (*Rnf130*) gene, which is found on chromosome 5 in humans [37] and 11 in mice (according to miRBase miRNA database). This provides a potential opportunity for transcriptional research concerning reproductive dysregulation induced by high-fat diets. miR-466c on the other hand is a rodent-specific nuclear microRNA encoded in the tenth intron of the Scm-like with four mbt domains 2 (*Sfmbt2*) gene [38]. mir-466c regulates *Vegfa* expression in a state of hypoxia in endothelial cells and suppresses *Runx2* to inhibit prostate cancer [38,39]. A microarray analysis performed to study microRNA-associated gene changes following spinal cord motor neuron degeneration in rats found a correlative relationship between miR-466c, which was downregulated from days 3 to 14 post-injury, and a GnRH signaling pathway which was markedly increased, starting from day 3 and most significant by day 14 post-injury [40]. This finding suggests a correlation between miR-466c repression and GnRH signaling, a finding corroborated by the results in our study. The fact that the overexpression of miR-466c blocked subsequent palmitate-mediated upregulation of *Gnrh* suggests that it is important for the effects of palmitate on *Gnrh.* Considering microRNAs target the 3′ UTR of genes, we investigated the possibility of direct binding to the *Gnrh* 3′UTR by looking for potential binding sites, and subsequent luciferase assays could be performed. Based on TargetScan predictions and poly(A) position profiling by sequencing (3P-seq) to assess the most probable length of the 3′UTR (approximately 100 bp) [41], the predicted miR-340 and miR-466c binding sites are likely outside of this *Gnrh* 3′UTR due to the sites being approximately 800 and 2900 base pairs downstream of *Gnrh*, respectively (Table 5). Whether these regions exist in the 3′UTR of the mouse *Gnrh* in our cell lines will need to be further validated using biotinylated microRNA pulldown assays, which would indicate if these microRNAs can actually target the 3′UTR of the *Gnrh* mRNA.

The transcriptional control of *Gnrh* has been well studied over the last couple of decades. The murine *Gnrh* gene promoter has a 173 bp AT-rich sequence, proximal to the transcription start site [3]. It is involved in hormonal regulation and contains binding sites for activating transcription factors, Oct-1 and Otx-2 homeodomain proteins, which are required for gene transcription [3]. Its proximal enhancer, a 300 bp sequence localized to the 5′ regulatory region, consists of binding motifs for CEBP-β, Oct-1 and GATA4. While studying the effects of palmitate, we therefore postulated that palmitate may be acting through these transcription factors to dysregulate *Gnrh*. Palmitate upregulated *Gata4* along with *Gnrh*, which suggests a possible mechanistic connection. Furthermore, microRNAs that increase (miR-340) and repress (miR-466c) *Gnrh* are equally changing *Gata4* in a manner that is congruent with their respective changes in *Gnrh* mRNA following mimic transfections. TargetScanMouse 7.1 predicts constituents of the mir-466 cluster to bind the 3′UTR of *Gata4*. Specifically, mir-466c-5p has a 7mer-1A seed match in the 569–602 bp region of the *Gata4* 3′ UTR, and the microRNA-mediated regulation of this transcription factor is planned for future studies. For this study, we were interested in how palmitate may pre- and post-transcriptionally regulate *Gnrh* expression through miRNAs.

A contextual-based function of microRNAs was proposed wherein a microRNA with its plethora of targets binds to a given target gene based on its relative expression level in the given cell [42]. Our results indicate that palmitate-mediated upregulation of *Gnrh* involves miR-340 repression. However, subsequent mimic transfection found that miR-340 upregulates *Gnrh*, as well as *Oct1* and *Cebpb*, which have both been found to be involved in the enhancer-specific repression of *Gnrh* by nitric oxide. Furthermore, miR-340 is known to differentially regulate cancer progression, acting as either a tumor suppressor or oncogene depending on the cancer cell [37]. These findings imply that the role of miR-340 is context-dependent. Therefore, miR-340 could potentially be involved in *Gnrh* mRNA repression under conditions of increased *Gnrh* expression or an inhibition of its repressors.

Our results indicate an antagonistic mRNA expression relationship between *Per2* and *Gnrh*. For instance, palmitate represses *Per2* while upregulating *Gnrh*, whereas the miR-466c mimic represses *Gnrh* while upregulating *Per2*. Gillespie et al. have shown that in the GT1-7 cell line, *Gnrh* is rhythmic and its rhythmicity mimics *Bma1l* expression, which is in antiphase of *Per2* [43]. These results suggest a potential role of miR-466c in modulating the circadian rhythmicity of *Gnrh* in the mHypoA-GnRH/GFP neurons that could be affected by palmitate exposure.

Despite being suppressed by palmitate, the roles of miR-503, miR-181b and miR-125a in the context of palmitate-mediated dysregulation of *Gnrh* in the mHypoA-GnRH/GFP neurons is not supported by the evidence presented herein. However, examining the past literature reveals their involvement in some aspects of reproduction. miR-503, for instance, is a potent ovarian microRNA with crucial functions in follicle formation, maturation, and the luteinization process [44,45,46]. Similar reproductive connections are noted for miR-181b, with its strong involvement in the sexual reproduction of boars [47,48,49], and miR-125a with differential expression levels from oogenesis to ovulation in mice [50], indicating their potential roles in fatty acid-induced reproductive dysfunction outside the scope of our experimental paradigm.

## 5. Conclusions

This study contributes to our understanding of the impact of nutritional choices on reproductive health. Our findings implicate specific miRNAs in the palmitate-mediated dysregulation of *Gnrh* and its associated transcription factors. Establishing this link between high-fat-diet consumption and reproductive neuropeptide expression through a mechanistic lens expands our knowledge of the consequences of increasing levels of palmitate. This knowledge could pave the way for microRNA-based therapeutics in metabolism-related diseases, targeting the mechanisms underlying diet-induced reproductive dysfunction in the context of obesity and infertility.

## Figures and Tables

**Table 1:** List of primers used for RT-qPCR.

Gene Name	Primer Sequence (5′ → 3′)	Amplicon Size (bp)
*Rpl7*	F: TCG CAG AGT TGA AGG TGA AG R: GCC TGT ACT CCT TGT GAT AGT G	114
*Gnrh*	F: CGT TCA CCC CTC AGG GAT CT R: CTC TTC AAT CAG ACT TTC CAG AGC	51
*Oct-1*	F: AGG AGC GAG TCA AGA TG R: CCA TTG GTT TGT GTG CCT GT	132
*Gata4*	F: AGA CAC CCC AAT CTC GAT ATG TT R: ATT GCA CAG GTA GTG TCC CG	117
*Cebpb*	F: CTG AGC GAC GAG TAC AAG ATG R: GAA CAA GTT CCG CAG GGT	186
*Otx2*	F: TGT TAC CAG CCA TCT CAA TC R: AGA GGC AGT TTG GTC CTT AT	118
*Chop*	F: TAT GAG GAT CTG CAG GAG R: CAG GGT CAA GAG TAG TGA AG	109
*Per2*	F: TCA TCA TTG GGA GGC ACA AA R: GCA TCA GTA GCC GGT GGA TT	135
*Bmal1*	F: GGG AGG CCC ACA GTC AGA TT R: GTA CCA AAG AAG CCA ATT CAT CAA	78

**Table 2:** List of microRNA primers used for RT-qPCR.

microRNA	Primer Sequence (5′ → 3′)
mmu-miR-125a-3p	F: CAG ACA GGT GAG GTT CTT G R: TCC AGT TTT TTT TTT TTT TTG GCT
mmu-miR-181b-5p	F: GCA GAA CAT TCA TTG CTG TC R: TCC AGT TTT TTT TTT TTT TTA ACC CA
mmu-miR-296-3p	F: GGA GGG TTG GGT GGA G R: GTC CAG TTT TTT TTT TTT TTT GGA GA
mmu-miR-328-3p	F: GCC CTC TCT GCC CTT C R: GGT CCA GTT TTT TTT TTT TTT TAC G
mmu-miR-340-5p	F: GCG CAG TTA TAA AGC AAT GAG R: GCT CCA GTT TTT TTT TTT TTT TAA TCA GT
mmu-miR-351-5p	F: GAG GAG CCC TTT GAG C R: GGT CCA GTT TTT TTT TTT TTT TCA
mmu-miR-466c-5p	F: GTG ATG TGT GTG TGC ATG T R: CAG GTC CAG TTT TTT TTT TTT TTT ATA TG
mmu-miR-485-5p	F: AGT CAT ACA CGG CTC TCC R: GCT CCA GTT TTT TTT TTT TTT TGA G
mmu-miR-503-5p	F: AGC AGC GGG AAC AGT R: CCA GTT TTT TTT TTT TTT TCT GCA GT
mmu-miR-574-3p	F: ACG CTC ATG CAC ACA C R: GTC CAG TTT TTT TTT TTT TTT GTG G
mmu-let-7k	F: GCA GTG AGG TAG GAG GT R: TCC AGT TTT TTT TTT TTT TTC ACA CA

**Table 4:** Murine microRNA candidates’ expression levels by percentages in hypothalamic models and predicted to be downregulated by palmitate based on microarray analysis.

	miRNA	Hypothalamus	mHypoE-46	mHypoA-59	PA Array Fold Change	Average CT
Not Conserved	mmu-miR-125a-3p	88	94	93	−1.56	26.5
	mmu-miR-181b-5p	97	97	97	−1.25	22.6
	mmu-miR-296-3p	88	96	93	−1.3	28.5
	mmu-miR-328-3p	98	93	94	−1.4	27.2
	mmu-miR-351-5p	87	95	96	−1.64	26.9
	mmu-miR-466c-5p	83	77	83	−1.43	28.9
	mmu-miR-503-5p,	72	89	92	−2.33	28.1
	mmu-miR-574-3p	89	93	96	−1.39	25.9
	mmu-let-7k	95	96	95	−1.38	35.9
Highly Conserved	mmu-miR-340-5p	75	48	39	−1.3 *	26.9
	mmu-miR-485-5p	90	83	78	−1.58	N/D
	mmu-mir-539-3p	36	24	10	1 *	N/D
	mmu-miR-876-5p	2	8	0	−1.01 *	N/D
	mmu-miR-224-5p	44	65	76	1.35	N/D

* not significantly changed, N/D = not done, PA = palmitate; average CT = cycle at threshold as assessed by qPCR.

**Table 5:** Binding sites of the candidate miRNAs in the *Gnrh* 5′UTR.

microRNA	Gnrh 3′UTR Binding Site
mmu-miR-340-5p	799–806
mmu-miR-351-5p	1241–1248
mmu-miR-503-5p	1918–1924
mmu-miR-125a-3p	2213–2219
mmu-miR-466c-5p	2915-2921
mmu-miR-181b-5p	4235–4242
hsa-miR-340-5p	65–71

## Data Availability

The data presented in this study are available on request from the corresponding author. The data are not publicly available due to privacy.
